# Association between Dietary Habits and Sperm Quality: Evidence from a Multicenter Fertility Study in Malaysia

**DOI:** 10.21315/mjms-09-2025-663

**Published:** 2026-02-28

**Authors:** Suriyani Muhamad, Nur Fatma Hasni Majid, Noor Salihah Zakaria

**Affiliations:** 1 Faculty of Business, Economics and Social Development, Universiti Malaysia Terengganu, Kuala Nerus, Terengganu, Malaysia; 2 Faculty of Food Science and Agrotechnology, Universiti Malaysia Terengganu, Kuala Nerus, Terengganu, Malaysia

**Keywords:** male fertility, sperm quality, dietary habits, socioeconomic factors, Malaysia

## Abstract

**Background:**

A range of factors, sociodemographic and lifestyle-related, are instrumental in shaping male fertility, including dietary practices. However, limited studies have examined these associations in the Malaysian context. This study investigates the connections between dietary habits and sperm quality among male patients attending fertility clinics.

**Methods:**

A cross-sectional design was used to examine male patients attending four fertility clinics in Peninsular Malaysia. Sociodemographic data, dietary habits, and sperm quality classifications (normal vs abnormal) were collected. Assessment of dietary consumption involved the use of validated food frequency questionnaires, while data on sperm quality were retrieved from patient’s medical records. Analysis of binary logistic regression was executed to assess food group consumption’s association with sperm quality, while controlling for household income and education level.

**Results:**

Sperm quality was significantly associated with education level (*P* = 0.001) and household income (*P* = 0.017), but not with age or body mass index (BMI). Higher fruit consumption was significantly associated with better sperm quality (*a*OR = 0.83; 95% CI: 0.73, 0.94; *P* = 0.004), while frequent fast-food intake was linked to poorer sperm quality (*a*OR = 1.55; 95% CI: 1.02, 2.35; *P* = 0.039). Vegetable, dairy, meat, caffeine, and sugar-sweetened beverage consumptions were not significantly associated with sperm quality.

**Conclusion:**

This study demonstrates the associations between dietary practices, socioeconomic variables, and male fertility. Higher fruit intake and socioeconomic status were associated with better sperm quality, whereas fast-food consumption was linked to poorer outcomes. The study reinforces the significance of diet and socioeconomic status in shaping male reproductive well-being.

## Introduction

Infertility, typically described as failing to become pregnant following 12 months of regular, unprotected sexual activity, remains a major public health concern worldwide, impacting an estimated 8% to 12% of reproductive-age couples (1). While infertility is often perceived as a female-centric issue, male factors contribute substantially, accounting for 20% to 30% of cases solely and up to 50% of cases as a whole (2). In Asia, male infertility is responsible for approximately 37% of infertility cases among couples attempting to conceive (3). Notably, male infertility research has revealed a downward trend in sperm quality among Asian males over the last 50 years (4), underscoring the urgency of addressing male reproductive health.

In Malaysia, a developing nation, fertility rates are on the decline, with the total fertility rate dropping to 1.7 births per woman in 2021, well below the global replacement level of 2.1 (5, 6). Replacement level fertility refers to the number of births needed for a population to maintain its size across generations. This decline, the third lowest among ASEAN countries, suggests potential underlying infertility challenges, including male infertility. However, comprehensive data on male fertility in Malaysia remain scarce, highlighting a critical gap in understanding the factors contributing to this trend.

Globally, male infertility is most often attributed to causes that include low sperm concentration, poor sperm motility, abnormal sperm morphology, and ejaculatory disorders. Emerging evidence indicates that male reproductive health is significantly influenced by lifestyle factors, particularly dietary practices (7). Maintaining a balanced and nutritious diet is not only vital for overall well-being but also crucial for supporting healthy sperm production, regulating hormonal function, and improving fertility potential. Conversely, consumption patterns rich in processed meats, saturated and trans fats, and highly processed sugars have been shown to be associated with elevated oxidative stress and sperm DNA damage, both of which can impair sperm quality (8). In contrast, eating patterns rich in antioxidants, omega-3 fatty acids, and anti-inflammatory compounds have been linked to better sperm parameters and improved fertility outcomes (9). These nutrients are commonly sourced from fish, whole grains, fruits, vegetables, and low-fat dairy products.

Despite this growing body of literature, most research has centred on Western populations, thus leaving limited insight into how dietary patterns influence male fertility in Southeast Asia, particularly Malaysia. The Malaysian diet is shaped by diverse cultural traditions and socioeconomic conditions, which may affect fertility outcomes differently than other regions. For example, rising fastfood consumption coupled with insufficient fruit and vegetable intake among Malaysian men may heighten infertility risks. Yet, empirical studies exploring these links in the Malaysian context are scarce (10). This study therefore, seeks to fill this gap by examining the connection between dietary practices and sperm quality involving male patients who attend fertility clinics in Peninsular Malaysia.

## Methods

### Study Design and Participants

The present cross-sectional research utilised a questionnaire-based survey approach. The sample comprised participants who were selected using a purposive sampling to ensure that they met predefined sperm quality parameters and other inclusion requirements. They were men aged between 20 and 60 years who were attending fertility clinics, actively trying to conceive with their partners, undergoing fertility treatment, and had completed a seminal fluid analysis as part of their evaluation. Men with incomplete medical records or lacking seminal fluid analysis data were excluded, as these details were critical for assessing sperm quality within the scope of this study. The sample was recruited through four fertility clinics that were willing to participate and grant access to relevant patient data. The sample comprised 1,068 male participants, exceeding the minimum required size. According to the Krejcie and Morgan formula, 283 respondents would be sufficient to test the study’s hypotheses (11).

### Data Collection

For data collection, a structured questionnaire was used, and its administration was through face-to-face and online formats. The online format was also employed to enhance participation and reduce barriers related to geographical distance. The questionnaire asked for detailed information in three key areas: (i) sociodemographic characteristics, (ii) dietary habits, and (iii) sperm quality. To cater to participants’ language preferences and ensure inclusivity, the questionnaire was available in both English and Malay. The Food Frequency Questionnaire, which assessed dietary habits, was interviewer-administered by trained research assistants to ensure clarity of questions and improve the accuracy of participant responses. The questionnaire was provided only to those meeting the criteria of eligibility and who gave written informed consent. The data collection period ran from November 2021 to June 2023.

### Research Instrument

#### Sociodemographic Characteristics

This study collected data on sociodemographic and anthropometric characteristics, including age, educational attainment, household income, and body mass index (BMI). Household income was classified according to the Household Income and Basic Amenities Survey Report 2019 (12) into three groups: the bottom 40% (B40) (< RM 4,850), the middle 40% (M40) (RM 4,850 to RM 10,959), and the top 20% (T20) (> RM 10,959). These categories were used to reflect the economic background of male patients seeking treatment at fertility clinics.

BMI was determined following the Asia-Pacific classification, which more accurately represents the physiological characteristics of Asian populations (13). Based on this framework, participants were categorised as underweight (BMI < 18.5 kg/m^2^), normal weight (BMI 18.5 to 22.9 kg/m^2^), or overweight/obese (BMI ≥ 23 kg/m^2^).

#### Dietary Habits Assessment

Dietary habits were assessed using a validated 159-item Food Frequency Questionnaire based on the Malaysian Adult Nutrition Survey 2014 (14). Participants recalled their intake of food over the previous month, reporting both the frequency of consumption and the portion sizes for every item of food that was listed. To improve the accuracy of dietary intake estimation, portion sizes were measured with a validated reference set and later converted to grams according to the standard portion sizes specified in the Malaysian Dietary Guidelines 2020 (15). The conversion of food frequency data into daily gram intakes followed a standardised equation consistent with the procedures applied in previous national nutrition surveillance studies (14):


$$\begin{array}{c} Amount\;of\;good\;consumer\;per\;day\;\left(\frac{g}{day}\right)\\ =frequency\;of\;intake\;(conversion\;factor)\times serving\;size\\ \times total\;number\;of\;serving\times weight\;of\;food\;in\;one\;serving \end{array}$$

The amount of food consumed per day was then converted into serving size per day (16):


$$Serving\;size=\frac{amount\;of\;food\;consumed\;per\;day}{weight\;of\;serving\;szie}$$

#### Sperm Quality

Male fertility was assessed through sperm quality evaluation, a standard procedure used to measure key indicators of male reproductive capacity. Semen analysis data were extracted from patients’ medical records at the participating fertility clinics. Seminal fluid analysis had been done by trained medical personnel in each clinic, in compliance with the World Health Organization (WHO) 2010 guidelines (17) to ensure standardised procedures across sites. In total, 304 semen samples were analysed, of which 140 were found normal and 164 abnormal. In reference to WHO criteria, abnormal semen parameters include a total sperm count below 39 million per ejaculate, a sperm concentration of less than 15 million per millilitre, and progressive motility under 32%. In contrast, normal sperm was identified by parameters that exceeded these established thresholds.

### Statistical Analysis

Data analysis involved the use of Statistical Package for the Social Sciences (SPSS) version 29. Descriptive statistics were used to calculate frequencies, percentages, means, and standard deviations. Categorical variables appeared as frequencies and percentages of the sample, with differences between sperm quality groups assessed through Pearson’s chi-square test (χ^2^) and Fisher’s exact test as needed. Continuous variables, in contrast, were expressed as means and standard deviations, with comparisons analysed through independent *t*-tests.

Binary logistic regression analysis was employed to assess whether dietary habits and sperm quality are associated. To address potential confounding effects, every variable found to have significance in the chi-square test were incorporated into the logistic regression analysis. Hence, two models were constructed: an unadjusted model, developed to examine the relationship between dietary intake and sperm quality without controlling for confounders, and an adjusted model, which incorporated confounding factors, including education level and household income. Multicollinearity among independent variables was assessed using the variance inflation factor, and all values were below 1.2, indicating no serious multicollinearity. Linearity of continuous variables with respect to the logit was confirmed using the Box-Tidwell test (*P* > 0.05). Model fit was evaluated with the Hosmer-Lemeshow test, the classification table, and the area under the Receiver Operating Characteristic (ROC) curve (AUC). Odds ratios (OR) accompanied by 95% confidence intervals (CI) were presented, with statistical significance established at *P* < 0.05.

## Results

### Characteristics of the Participants

Table 1 outlines participants’ sociodemographic profiles and examines their association with sperm quality. Education level was significantly associated with sperm quality, as indicated by the *P*-values (*P* = 0.001). A relatively greater proportion of participants with tertiary education was observed in the normal sperm quality group (19.7%) than in the abnormal group (13.8%). Conversely, participants with secondary education were more concentrated in the abnormal sperm quality group (39.8%) compared with the normal group (25.3%).

Similarly, household income showed a significant association with sperm quality (*P* = 0.017). Among participants with normal sperm quality, higher proportions were observed in the middle-income (M40, 20.4%) and high-income (T20, 9.2%) categories, while a smaller proportion was in the lower-income group (B40, 16.4%). In contrast, the abnormal sperm quality subset comprised a greater proportion of those from the B40 group (24.7%) and slightly more from the M40 group (24.3%), with only 4.9% from the T20 category.

On the other hand, age and BMI were not significantly associated with semen quality, as indicated by their respective *P*-values (age: *P* = 0.723; BMI: *P* = 0.268), both of which exceed the conventional threshold for statistical significance (*P* < 0.05). Most participants were aged less than 40 years, with a slightly higher proportion of abnormal semen quality observed in this age group (41.8%) compared to those with normal semen quality (34.9%). Regarding BMI, most participants were classified as overweight or obese, accounting for 33.6% of those with normal semen quality and 34.5% of those with abnormal semen quality. A smaller proportion had a normal BMI, while only a few participants were underweight.

### Dietary Habits Intake by Food Group

The dietary habits and significant differences in food group intake between participants with different types of sperm quality were presented in Table 2. A statistically significant difference in fruit consumption existed for the two groups of sperm quality (*P* < 0.001). Participants with normal sperm quality reported a higher mean fruit intake (2.97 ± 2.67) compared to those with abnormal sperm quality (1.89 ± 1.99). In contrast, fast-food consumption also demonstrated a significant difference (*P* = 0.024). Participants with abnormal sperm quality were found to have a higher mean intake of fast food (0.72 ± 0.70) compared to those with normal sperm quality (0.62 ± 0.55).

For other food groups, no statistically significant differences were observed. Vegetable consumption, although higher in the normal sperm quality group (6.73 ± 4.41) compared to the abnormal group (5.10 ± 4.04), did not reach statistical significance (*P* = 0.103). Similarly, dairy product consumption (normal: 0.74 ± 0.70; abnormal: 0.61 ± 0.73; *P* = 0.660), meat consumption (normal: 1.87 ± 1.24; abnormal: 1.94 ± 1.41; *P* = 0.353), caffeine consumption (normal: 1.57 ± 1.21; abnormal: 1.73 ± 1.31; *P* = 0.139), and sugar-sweetened beverage consumption (normal: 0.73 ± 0.82; abnormal: 0.60 ± 0.79; *P* = 0.438) did not show significant associations with sperm quality.

### Association between Dietary Practice and Sperm Quality

The findings in Table 3 indicate that fruit consumption is significantly associated with better sperm quality (OR = 0.81; 95% CI: 0.71, 0.92; *P* = 0.002) even after adjustment (OR = 0.83; 95% CI: 0.73, 0.94; *P* = 0.004). These results suggest that participants who consume more fruits are less likely to experience poor sperm quality, even after adjusting for socioeconomic factors. Vegetable consumption was also marginally significant in the crude model (OR = 0.94, 95% CI: 0.88, 0.99; *P* = 0.045); however, after household income and education level were adjusted, the association weakened and lost statistical significance (*a*OR = 0.94; 95% CI: 0.88, 1.00; *P* = 0.051). This suggests that vegetable intake may contribute to improved sperm quality, though its impact may not be as strong as fruit consumption.

In contrast, fast-food consumption was found to be significantly linked with poorer sperm quality (*a*OR = 1.55; 95% CI: 1.02, 2.35; *P* = 0.039). These results suggest that participants who regularly consume fast food have a notably higher risk of presenting abnormal sperm parameters, even when considering other factors, namely income and education. This highlights the potential detrimental effect of unhealthy dietary habits, especially those rich in processed foods and unhealthy fats, on male reproductive health.

Other dietary variables, including the intake of dairy products, meat, caffeine, and sweet drinks, did not have a significant association with sperm quality in either the unadjusted or adjusted analyses. For instance, dairy product consumption had a crude OR of 0.89 (95% CI: 0.63, 1.26) and an adjusted OR of 1.03 (95% CI: 0.71, 1.48), indicating no meaningful effect on sperm quality. Similarly, caffeine intake (*a*OR = 1.15, 95% CI: 0.94, 1.41) and sugar-sweetened beverage consumption (*a*OR = 0.82, 95% CI: 0.59, 1.13) failed to indicate statistically significant relationships with sperm quality.

## Discussion

Male infertility contributes substantially to the global burden of reproductive health issues, accounting for nearly 50% of overall infertility cases. Among those factors influencing male reproductive health, lifestyle and dietary behaviours, alongside socioeconomic status, have gained increasing recognition as important determinants of semen quality. Consistent with this understanding, the present study’s results have shown that there are significant associations between dietary habits and sperm quality among male patients attending fertility clinics in Peninsular Malaysia. Specifically, higher fruit consumption was positively correlated with normal sperm quality, whereas frequent fast-food consumption had a link to poorer semen parameters. These outcomes demonstrate that dietary patterns play a critical role in shaping male reproductive health, even after controlling for socioeconomic variables such as education and household income.

Socioeconomic status, particularly education and income levels, also showed a notable impact on sperm quality. Participants who possess a higher level of education and greater household income had a greater likelihood of exhibiting normal sperm parameters compared to those with abnormal results. This association may reflect increased health literacy and the adoption of healthier lifestyle behaviours, which in turn support improved reproductive health. Greater education may enhance awareness of the importance of nutrition, exercise, and overall wellness, fostering behaviours that positively affect sperm quality. Similarly, higher income levels may enable access to better-quality food, healthcare, and fertility treatments, all of which can contribute to more favourable reproductive outcomes. These discoveries support findings in previous studies documenting links between socioeconomic factors, particularly education and income, and sperm quality.

One of the studies found a significant decline in sperm quality among Malaysian men with lower educational levels, particularly those with only secondary education or below (10). This was attributed to limited fertility knowledge and unhealthy lifestyle practices. Similarly, a study in the US reported that males possessing only a high school education had a greater likelihood of abnormal semen quality, whereas those with at least a bachelor’s degree showed improved parameters (18). These studies reinforce the hypothesis that higher education level contributes to better reproductive health awareness and healthier lifestyle choices, ultimately leading to improved sperm quality.

In terms of income, the current findings are in line with van Wijk (19), who reported a strong, positive association between income and fertility, particularly among men. Financial resources may enable healthier diets and timely access to medical care, both of which support reproductive function. However, contrary findings from Muhamad et al. (10) have highlighted the absence of a significant association concerning income and semen quality for a Malaysian sample. This discrepancy may stem from differences in sample characteristics or the relatively healthier lifestyle practices maintained by lower-income participants in the previous study, which may have mitigated income-related fertility disparities.

However, it was observed that BMI and sperm quality did not have a significant association. This finding contrasts with the result of Altimimi and Almurshdi (20), who reported that advancing age and elevated BMI were associated with deteriorated sperm parameters. The lack of significant associations in our study may be explained by the relatively young age distribution of participants and the narrow variability in BMI categories. Most participants were under 40 years of age, possibly reducing the age-related impact on semen quality. Moreover, even with overweight or obese participants, the distribution of BMI between those with normal and abnormal sperm quality was not significantly different, suggesting BMI alone may not be a decisive factor without considering other metabolic or lifestyle variables.

Integrating these findings with dietary habits provides a clearer understanding of how lifestyle and socioeconomic conditions intersect to influence male fertility. Participants with normal sperm quality reported significantly higher fruit consumption (*P* < 0.001), and logistic regression confirmed a strong inverse relationship between fruit intake and the likelihood of abnormal sperm quality, even after the adjustment for income and education (*a*OR = 0.83; 95% CI: 0.73, 0.94; *P* = 0.004). These findings concur with those in previous studies, which showed that fruits, abundant in antioxidants such as vitamin C, folate, and polyphenols, help protect sperm from oxidative damage and DNA fragmentation (21–23). Since oxidative stress is a major factor impairing sperm function, diets rich in antioxidants may reduce this harmful effect and improve sperm motility, morphology, and concentration.

Besides, vegetable consumption showed a marginally significant crude association with improved sperm quality (OR = 0.94; 95% CI: 0.88, 0.99; *P* = 0.045), lost statistical significance in the adjusted model (*a*OR = 0.94; *P* = 0.051). This suggests that the apparent benefit of vegetable intake may be confounded by underlying socioeconomic status. It is possible that individuals with higher education and income not only consume more vegetables but also engage in multiple health-promoting behaviours, making it difficult to isolate the specific contribution of vegetables alone. Nevertheless, vegetables provide vitamins A, C, E, and fibre, nutrients that are known to support antioxidant defence mechanisms and sperm function (7).

Conversely, frequent fast-food consumption was significantly associated with poorer sperm quality (*a*OR = 1.55; 95% CI: 1.02, 2.35; *P* = 0.039), even after adjusting for confounders. Fast food is often high in trans fats, sodium, and preservatives, which contribute to oxidative stress, systemic inflammation, and hormonal disruption, all of which negatively affect sperm parameters (24, 25). Prior research supports these findings, strongly suggesting that Western dietary patterns, which typically include large amounts of processed and fatty foods, are detrimental to male fertility (8).

Meanwhile, other dietary components, including dairy products, meat, caffeine, and beverages containing sugar, had no significant association with sperm quality in this study. Several factors may help explain these neutral findings. First, the reported intake levels for these foods may not have been sufficiently high or varied among participants to produce measurable effects on semen parameters. Second, although the Food Frequency Questionnaire is widely used to assess habitual dietary intake, it is subject to recall bias, and participants may under-report or over-report certain food items, particularly those perceived as unhealthy. Such misreporting could attenuate true associations and contribute to non-significant results. Third, the Malaysian dietary context, where these items are often consumed in moderation or incorporated into mixed dishes, may further dilute any independent effect on sperm quality. These results align with existing literature. For example, Wang et al. (26) reported that general dairy intake and sperm parameters did not have a significant association, although they noted a possible risk from high dairy fat intake. Similarly, Meldgaard et al. (27) found no strong relationship between beverages containing sugar and sperm quality among young men. Findings on caffeine remain mixed in the literature, with some studies suggesting adverse effects (28) and others reporting no significant associations (29), aligning with the neutral results in the current study.

Nevertheless, the findings also reveal that dietary intake and sperm quality had a more pronounced association after controlling for education level and household income. This highlights the potential role of socioeconomic factors in shaping dietary behaviours, which in turn may affect reproductive health outcomes. Men with higher education levels may have greater health literacy, enabling them to adopt healthier lifestyles that contribute to improved sperm quality. These results emphasise the need to promote nutritious dietary habits as part of comprehensive public health strategies aimed at enhancing male reproductive health, especially within lower socioeconomic groups.

This study has several strengths. Firstly, it utilises established and validated questionnaires from previous Malaysian studies to assess dietary practices, ensuring cultural relevance and methodological reliability. This approach provides a more precise reflection of dietary habits relevant to the Malaysian population. Secondly, the study highlights the significance of healthy eating habits, particularly for male infertility patients, addressing a crucial but underexplored aspect of male reproductive health in Malaysia. By emphasising the role of diet in male fertility, the findings offer meaningful knowledge regarding an area that has received limited attention in local research.

However, certain limitations must be acknowledged. First, access to fertility clinics with comprehensive male fertility data was restricted, as many clinics had only a small number of male fertility cases, limiting the scope of data collection. Secondly, obtaining approval to use patient data posed a challenge due to confidentiality concerns, resulting in the study being conducted in only four fertility clinics. Thirdly, male fertility remains a sensitive topic, which was evident in the difficulty of obtaining informed consent and convincing male patients to participate in the survey. Additionally, the use of purposive sampling may introduce selection bias, and the study sample may not fully represent the broader population, which could affect the generalizability of the results. Finally, the cross-sectional design of this study limits the ability to establish causal relationships between dietary habits and sperm quality, as the findings only reflect associations. Despite these limitations, the study provides valuable preliminary insights into dietary factors associated with sperm quality, which may inform future large-scale and longitudinal research in this area.

## Conclusion

In conclusion, this study provides evidence that, among men who frequent fertility clinics in Peninsular Malaysia, dietary habits are significantly linked to sperm quality. Specifically, higher fruit intake was associated with improved sperm quality, whereas frequent fast-food consumption correlated with poorer sperm parameters, even after controlling for education level and household income. These results suggest that it is important to adopt healthy diets and enhance socioeconomic support in order to address male fertility issues. However, future studies should also investigate other modifiable lifestyle variables, like physical activity, psychological stress, and environmental exposures, to be able to develop more comprehensive fertility interventions.

## Figures and Tables

**Table 1:** Sociodemographic characteristic

Variables	Sperm quality		*P*-value

	Normal (*n* = 140) *n* (%)	Abnormal (*n* = 164) *n* (%)	
**Age**			

Less than 40 years	106 (34.9)	127 (41.8)	0.723^a^
40 years and above	34 (11.2)	37 (12.2)	

**Educational levels**			

Primary education	3 (1.0)	1 (0.3)	^**^ **0.001** b
Secondary education	77 (25.3)	121 (39.8)	
Tertiary education	60 (19.7)	42 (13.8)	

**Household income**			

B40	50 (16.4)	75 (24.7)	^*^ **0.017** a
M40	62 (20.4)	74 (24.3)	
T20	28 (9.2)	15 (4.9)	

**Body mass index**			

Underweight	1 (0.3)	2 (0.7)	0.268^b^
Normal	37 (12.2)	57 (18.8)	
Overweight or obese	102 (33.6)	105 (34.5)	

a Pearson chi-square test;

b Fisher’s exact test;

*P*-value = significant value at ^*^P < 0.05 and ^**^P < 0.01

**Table 2:** Dietary habits intake by food group

Food group	Semen quality Mean (serving/day) ± SD		*t*	*P*-value

	Normal (*n* = 140)	Abnormal (*n* = 164)		
Fruits	2.97 ± 2.67	1.89 ± 1.99	3.938	**< 0.001** ^**^
Vegetables	6.73 ± 4.41	5.10 ± 4.04	3.357	0.103
Dairy products	0.74 ± 0.70	0.61 ± 0.73	1.541	0.660
Meat	1.87 ± 1.24	1.94 ± 1.41	–0.499	0.353
Fast food	0.62 ± 0.55	0.72 ± 0.70	–1.308	**0.024** ^*^
Caffeine	1.57 ± 1.21	1.73 ± 1.31	–1.055	0.139
Sugar-sweetened beverage	0.73 ± 0.82	0.60 ± 0.79	1.468	0.438

*t*-value = independent *t*-test significant at ^*^P < 0.05, ^**^P < 0.001; SD = standard deviation

**Table 3:** Association between dietary practice and sperm quality using binary logistic regression analysis

Food group	OR (95% CI)	*P*-value	*a*OR (95% CI)	*P*-value
Fruits consumption	0.81 (0.71, 0.92)	**0.002** ^*^	0.83 (0.73, 0.94)	**0.004** ^*^
Vegetable consumption	0.94 (0.88, 0.99)	**0.045** ^*^	0.94 (0.88, 1.00)	0.051
Dairy products consumption	0.89 (0.63, 1.26)	0.530	1.03 (0.71, 1.48)	0.863
Meat consumption	1.12 (0.92, 1.36)	0.237	1.09 (0.89, 1.32)	0.379
Fast-food consumption	1.52 (1.01, 2.29)	**0.043** ^*^	1.55 (1.02, 2.35)	**0.039** ^*^
Caffeine consumption	1.13 (0.93, 1.37)	0.204	1.15 (0.94, 1.41)	0.159
Sugar-sweetened beverage consumption	0.90 (0.65, 1.25)	0.553	0.82 (0.59, 1.13)	0.236

Significant value at *P* < 0.05; OR = crude odds ratio for all of the variables above; *a*OR = adjusted odds ratio for all of the variables above including household income and educational level; CI = confidence interval
